# The effect of respiratory motion on electronic portal imaging device dosimetry

**DOI:** 10.1002/acm2.12541

**Published:** 2019-02-05

**Authors:** Andrew L. Fielding, Jessica Benitez Mendieta, Sarah Maxwell, Catherine Jones

**Affiliations:** ^1^ Science and Engineering Faculty Queensland University of Technology (QUT) Brisbane Qld Australia; ^2^ Department of Radiation Oncology Princess Alexandra Hospital Brisbane Qld Australia

**Keywords:** dosimetry, EPIDs, motion, radiotherapy

## Abstract

There is an increasing need to develop methods for *in vivo* verification of the delivery of radiotherapy treatments. Electronic portal imaging devices (EPID's) have been demonstrated to be of use for this application. The basic principle is relatively straightforward, the EPID is used to measure a two‐dimensional (2D) planar exit or portal dose map behind the patient during the treatment delivery that can provide information on any errors in linear accelerator output or changes in the patient anatomy. In this paper we focused on the effect of intra‐fraction motion, particularly respiratory motion, on the measured 2D EPID dose–response. Measurements were made with a breast phantom undergoing one‐dimensional (1D) sinusoidal motion with a range of amplitudes (0.5, 1.0, and 1.5 cm) and frequencies (12, 15, and 20  cycles/min). Further measurements were made with the phantom undergoing breathing sequences measured during patient planning computed tomography simulation. We made use of the quadratic calibration method that converts the EPID images to a surrogate for dose, equivalent thickness of Plastic Water^®^. Comparisons were made of the 2D thickness maps derived for the different motions compared to the static phantom case and the resulting dose difference analyzed over the “breast” region of interest. A 2D gamma analysis within the same region of interest was performed of the motion images compared to static reference image. Comparisons were made of 1D thickness profiles for the moving and static phantom. The 1D and 2D analyses show the method to be sensitive to the smallest motion amplitude of 0.5 cm tested in the phantom measurements. The results using the phantom demonstrate the method to be a potentially useful tool for monitoring intra‐fraction motion during the delivery of patient radiotherapy treatments as well as more generally providing information on the effects of motion on EPID based *in vivo* dosimetric verification.

## 1 INTRODUCTION

Technological advances in the planning and delivery of radiation therapy has led to an increase in complexity of patient treatments.^1^ This increase in complexity has led to an increased sensitivity to uncertainties and errors in the treatment process and a subsequent need for verification of the delivered treatment.^2^ Electronic portal imaging devices (EPIDs) have been shown to be suitable for *in vivo* dosimetric verification of treatments.^2^, ^3^, ^4^, ^5^, ^6^ The concept for using two‐dimensional (2D) flat panel EPIDs for pretreatment and treatment time verification is conceptually straightforward and can be performed in one of two ways. A 2D integrated measurement of the delivered dose is made during radiation delivery and then compared with a reference 2D dose map calculated (or measured) at the EPID plane. Alternatively, the 2D EPID dose measurement can be back‐projected into a CT‐based model of the patient and compared with a reference dose distribution in the same CT‐based patient model.^7^ The CT model can be derived from the treatment planning CT or for a more accurate prediction be based on a treatment time cone beam CT. EPIDs have been shown in a number of studies to be valuable for dosimetric verification of breast radiotherapy treatments.^8^, ^9^, ^10^, ^11^, ^12^, ^13^ As already stated although this appears to be conceptually straightforward, the implementation of a solution with high accuracy is nontrivial such that the technique is still not in widespread routine clinical use.

A technique for using EPID‐based measurements of radiological thickness for verifying the delivery of radiotherapy treatments has been previously demonstrated.^3^, ^14^, ^15^ This was an extension of the quadratic calibration method that has been used for designing compensators and IMRT fields for tangential breast radiotherapy treatment fields.^16^, ^17^, ^18^ The technique was shown to be a reliable means of directly relating measured EPID images to a reference Monte‐Carlo EPID simulation, using the radiological or poly(methyl)methacrylate equivalent thickness as a surrogate for dose. It was shown to be suitable for verifying treatment delivery and identifying changes in the treatment field, patient position, and target location as well as patient physical thickness.

Differences between delivered and planned dose distribution can have a number of sources including radiation delivery errors, changes in detector response, and changes in patient anatomy which would change the transmitted dose reaching the portal dosimeter. Potential radiation delivery errors and intra‐ and inter‐fractional changes in the patient anatomy will be detrimental to the treatment outcome and are the reason *in vivo* treatment verification is highly desirable.^19^, ^20^ EPID dosimetry is considered to be the favored modality for *in vivo* dosimetry going into the future and it has been shown to be effective for detecting the various types of errors encountered in the radiotherapy treatment.^20^, ^21^, ^22^

If EPID dosimetry is going to be more widely used in the clinical setting it is important that the sources of potential differences between measured and reference dose to the EPID are understood and characterized. One of these sources is intra‐fraction respiratory motion that would be expected to cause a difference between the predicted and measured signal in the EPID. This paper investigates the effect of respiratory motion on measured EPID images converted to radiological thickness maps. An emphasis is placed on the larger motion amplitudes that the literature suggests can occur in patients undergoing thoracic radiotherapy.^23^, ^24^, ^25^ The study aims to investigate how different magnitudes of regular and irregular patient motion sequences manifest in the EPID images when calibrated for radiological thickness and to determine if the method could be suitable for simple and efficient monitoring of intra‐fraction patient motion and compliance with breath — hold techniques. The work will also provide important information on the effects of motion on EPID dosimetry techniques.

## 2 MATERIALS AND METHODS

### 2.A Calibration

Two sets of measurements were performed using an Elekta Precise and an Elekta Agility linear accelerator in 6 MV photon mode operating at a nominal dose rate of 600 MU/min. The method described in this paper is independent of the accelerator and therefore when presenting and discussing this work the accelerator will not be specified unless it is considered relevant. Images were obtained using an iView GT amorphous silicon electronic portal imaging device mounted on the linear accelerator gantry. Images were acquired using the Heimann Imaging Software (available on the iView acquisition computer) as this offered greater flexibility than the standard iView acquisition software. The quadratic calibration method makes use of a series of calibration images of different thickness of water equivalent material, in this work slabs of Plastic Water^®^ were used (Computerized Imaging Reference Systems, Inc, Norfolk, VA, USA). Seven calibration images, with a flood radiation field size of 26 × 26 cm^2^ (defined at the isocentre plane and corresponding to 41 × 41 cm^2^ at the EPID plane in order to calibrate every pixel in the detector) were obtained for Plastic Water thicknesses of 2, 4, 7, 11, 16, and 21 cm, as well as an open‐field image with no phantom material in the field; each calibration measurement required the phantom to be setup iso‐centrically on the treatment couch with the range of thicknesses chosen to represent the typical separation of the patient's breast. Each calibration image was acquired with an exposure of 100 MU and 60 frames (frame integration time was 430 ms) with a frame averaged image being generated on completion of each acquisition. An offset image *I*_*dark*_ *(x,y)* was also obtained and used for correcting for background dark current signal in the raw images, *I*_*raw*_ *(x,y)* such that the resulting image *I(x,y)* was given by

(1) $$I\left(x,y\right)=I_{\mathrm{raw}}\left(x,y\right)-I_{\mathrm{dark}}\left(x,y\right)$$

The quadratic calibration method, described in more detail in Ref. 3, 26, was applied to the seven calibration transmission images, and a set of calibration coefficients, *α*(*x*,*y*) and *β*(*x*,*y*), was derived for each pixel in the detector. The calibration coefficients, obtained using a least squares polynomial fit, relate the intensity signal, *I*(*x*,*y*), measured in each detector pixel to equivalent Plastic Water^®^ thickness, *t*_PW_(*x,y*) through the expression,

(2) $$I\left(x,y\right)=I_{0}\left(x,y\right)e^{-\alpha \left(x,y\right)t\left(x,y\right)-\beta \left(x,y\right)t^{2}\left(x,y\right)}$$

The quadratic term (*t* ^*2*^) in Equation (2) is introduced to account for spectral variations in the linear accelerator photon beam and beam hardening in the object which cause the signal recorded by a detector pixel (*x,y*) to deviate from a simple exponential function of object thickness. Equation (2) can then be inverted in order to convert subsequent images of any object to equivalent Plastic Water^®^ thickness maps, *t*_PW_(*x,y*). An iterative algorithm is used to incorporate corrections for differences in field size between the calibration field size and the treatment field size, and scatter.^3^, ^16^, ^17^, ^18^ The first estimate of the thickness map *t*_PW_(*x,y*) is calculated using

(3) $$t_{\mathrm{PW}}\left(x,y\right)=\frac{-\alpha \left(x,y\right)\pm \sqrt{\alpha {\left(x,y\right)}^{2}-4\beta \left(x,y\right)ln\left[I\left(x,y\right)/I_{0}\left(x,y\right)\right]}}{2\beta (x,y)}$$

A new estimate for the image pixel intensity, corrected for field size and scatter, is then calculated using

(4) $$I_{i+1}\left(x,y\right)=F.\left(\frac{1+{\text{SPR}}_{\mathrm{ref}}\left(x,y\right)}{1+{\text{SPR}}_{\mathrm{treat}}\left(x,y\right)}\right)\cdot I_{i}\left(x,y\right)$$

where *I*_i_(*x,y*) is the pixel intensity from the original uncorrected image or previous iteration of the correction, F denotes an open‐field output factor, equal to the ratio of the open‐field EPID signal, *I*_open_(*x, y*), at the treatment field size to the calibration field size *I*_calib_(*x, y*). SPR_ref_ (*x*,* y*) is the scatter‐to‐primary ratio for the calibration measurements and SPR_treat_(*x*,* y*) is the scatter‐to‐primary ratio for the breast phantom measurements (treatment field size). The first order approximation used for calculating the SPR is given by Ref. 27

(5) $$\text{SPR}\left(x,y\right)=k_{0}\;A\;t\left(x,y\right)$$

where *A* is the field size (area) at the isocentre plane, and *t*(*x, y*) is the thickness of the object (calibration phantom or breast phantom) and *k*_0_ is a parameter with a value of 1.93 × 10^−5^/cm^3^ to account for system geometry and the Plastic Water^®^ calibration phantom electron density.^3^, ^27^ An updated *t*_PW_(*x, y*) (using Equation (3)] is then calculated using the new *I*_*i*+1_ (*x*,* y*) of Equation (4).This correction algorithm for calculating the thickness image *t*_PW_(*x, y*) was repeated and found to converge after five iterations.

### 2.B Treatment fields

The treatment fields were planned using the Pinnacle treatment planning system (TPS; Phillips Healthcare, Best, Netherlands). CT data of the CIRS thorax phantom (Computerized Imaging Reference Systems, Inc, VA, USA) was imported into the TPS and a parallel opposed wedged pair treatment was designed for the isocentric phantom geometry. Field sizes for the Elekta Synergy and Agility setups were asymmetric 14 × 8 cm^2^ and 16 × 8 cm^2^ respectively delivered from an anterior oblique gantry angle of 68 degrees. The wedge angle of 37° was generated using a universal wedged component of 153.6 MU and an open‐field component of 55.4 MU.

### 2.C Motion simulation

**Figure 1:**
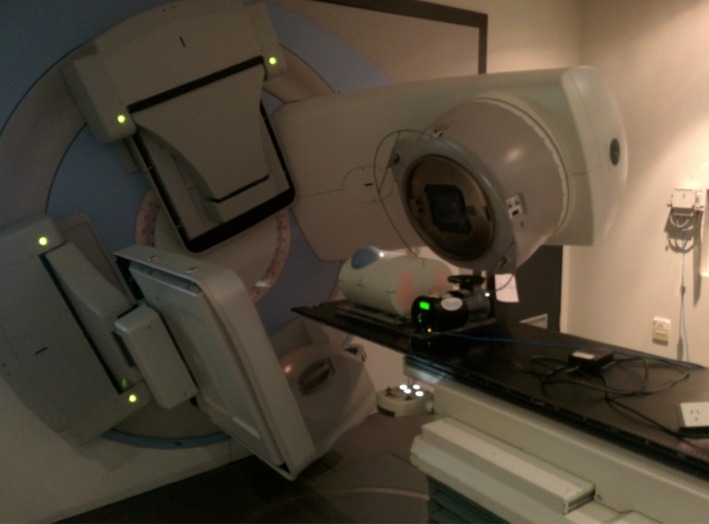
The experimental setup for simulating the effects of motion on breast radiotherapy. The direction of one‐dimensional motion was along the longitudinal couch direction.

The anterior oblique wedged field was delivered to the CIRS thorax phantom (including a breast attachment) that was fixed on the CIRS programmable motion platform. Figure 1 shows a photograph of the experimental setup of the linear accelerator, EPID, phantom and motion platform. Measurements were performed with a static phantom and with the same phantom undergoing different motion sequences. The static phantom measurement was repeated three times to test reproducibility. Table 1 lists the different motion sequences that were used. The six patient respiratory motion sequences used in this study were obtained from Varian RPM measurements during CT treatment planning simulation scans. The motion was then replicated by the CIRS programmable motion platform with radiation delivery starting at an arbitrary point in the respiratory cycle, that is*,* no synchronization between the radiation delivery and the motion sequence was performed. Repeat irradiations were performed for each motion sequence to check for any synchronization or interplay effects. The same setup was used for measurements performed on the Elekta Synergy and Agility linear accelerators.

**Table 1:** Summary of the different motion sequences used for the study

	Elekta synergy, iView GT	Elekta agility, iView GT
Sinusoidal motion	Amplitude: 0.5 cm Frequency: 12, 15, 20 cycles/min	Amplitude: 1.5 cm Frequency: 18 cycles/min
	Amplitude: 1.0 cm Frequency: 12, 15, 20 cycles/min	
	Amplitude: 1.5 cm Frequency: 12, 15, 20 cycles/min	
Patient motion	1 patient, 2 repeats	5 patients, 2 repeats each

### 2.D Image analysis

A threshold technique was first used to create a mask representing the radiation field in the images. The boundary pixels (field edge) of the mask were determined using an implementation of a “chain‐code” algorithm.^28^, ^29^ EPID images of the breast phantom were converted to 2D thickness maps using the quadratic calibration technique described in Section 2.A. The “breast” outline in the static thickness image was manually contoured to create a region of interest (ROI) and the image pixels within the ROI determined using the POLYFILLV function of IDL (Interactive Data Language, Harris Geospatial Solutions, Inc, Broomfield, CO, USA).

A comparison of the thickness maps with the phantom undergoing motion to the thickness map obtained for the static case was performed. Comparison was performed in 2D and for 1D profiles taken through the 2D thickness maps. The 2D thickness maps were also converted back into Intensity maps I(*x,y*), using Equation (2), and *α* and *β* values of 0.05/cm and 1 × 10^−4^ /cm respectively so that the dose difference, *I*_motion_(*x*,* y*) − I_static_(*x*,* y*), could be analyzed. Dose differences between the static and motion images were analyzed for individual pixels within the ROI. The spatial accuracy of dose delivery is also important such that small changes in high‐dose gradient regions (or high thickness gradients) could lead to significant dose differences. Therefore, the dose differences in the ROI between static and moving images were also quantitatively assessed using a gamma analysis that combines the distance to agreement criteria with dosimetric difference (local dose difference was used in this analysis), for a range of different pass criteria including 5%/5 mm, 3%/3 mm, 2%/2 mm, and 1%/1 mm.^30^

## 3 RESULTS

**Figure 2:**
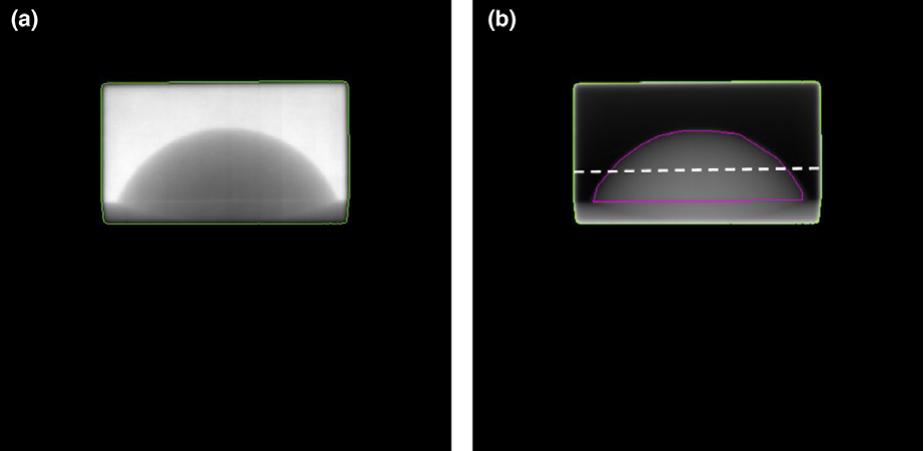
(a) Image of static breast phantom and (b) Thickness map after quadratic calibration. The radiation field edge is shown as green line and segmented “breast” region of interest as magenta line. The horizontal dashed white line indicates the approximate location of one‐dimensional profiles in subsequent figures.

Figure 2(a) shows the measured treatment field EPID image of the static CIRS breast phantom. The image was calibrated using the quadratic calibration method and the resulting Plastic Water^®^ equivalent thickness map, *t*_PW_(*x*,* y*) is shown in Fig. 2(b). Figure 2(b) also shows the field edge and contoured “breast” ROI. As well as providing a measurement of equivalent Plastic Water^®^ thickness, the quadratic calibration method also improves image quality, and has been shown to be superior to the standard pixel by pixel gain corrections employed in clinical MV imaging.^26^

**Figure 3:**
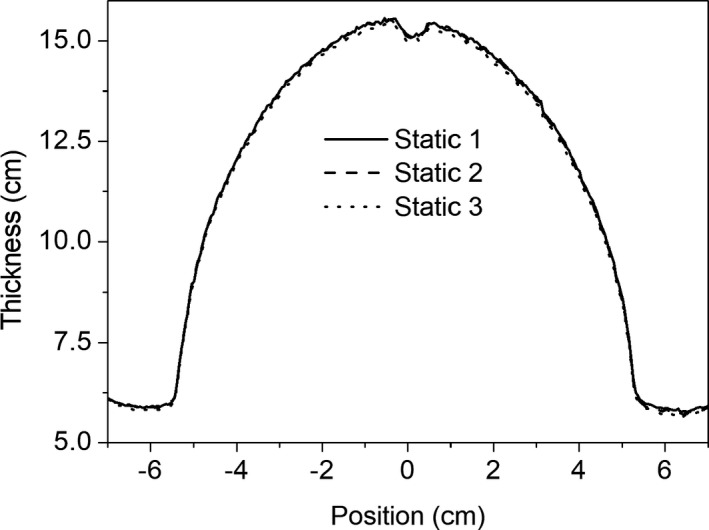
Measured profiles through the quadratically calibrated thickness map for the static breast phantom, repeated three times to demonstrate reproducibility of the method.

**Figure 4:**
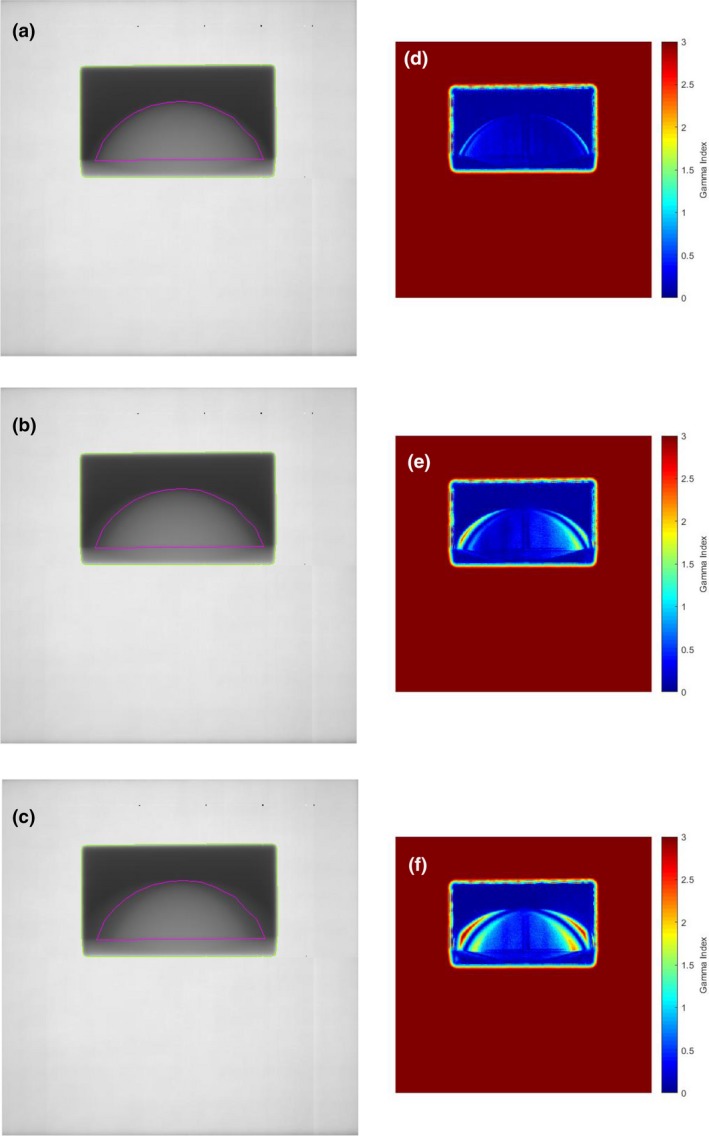
Calibrated thickness maps of the breast phantom undergoing sinusoidal motion with a frequency of 12 cycles/min and amplitudes of (a) 0.5 cm, (b) 1.0 cm, and (c) 1.5 cm. Green line indicates the radiation field edge and magenta indicates the segmented “breast” region of interest. Results of a two‐dimensional gamma analysis of the target motion dose images calculated from thickness images in (a), (b), and (c) compared to the reference static dose image are shown in (d), (e), and (f) for gamma criteria 2%/2 mm.

**Figure 5:**
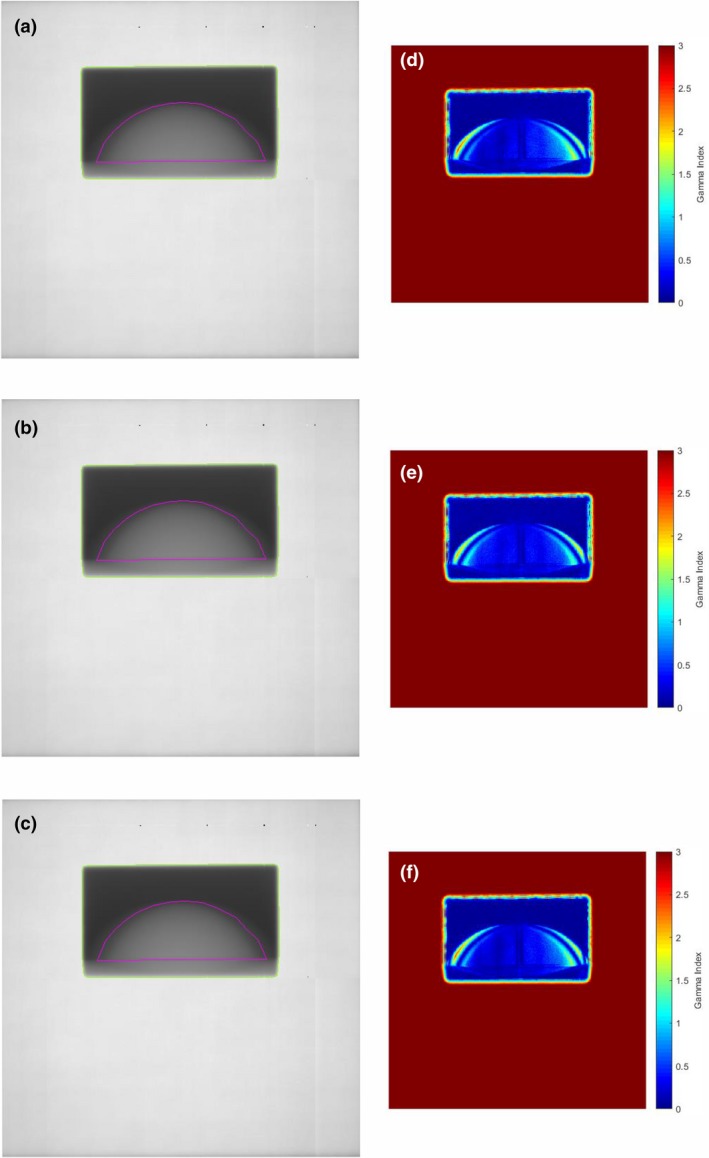
Calibrated thickness maps of the breast phantom undergoing sinusoidal motion with an amplitude of 1 cm and frequencies of (a) 12 cycles/min, (b) 15 cycles/min, and (c) 20 cycles/min. Green line indicates the radiation field edge and magenta indicates the segmented “breast” region of interest. Results of a two‐dimensional gamma analysis of the target motion dose images calculated from the thickness images in (a), (b), and (c) compared to the reference static dose image are shown in (d), (e), and (f) for gamma criteria of 2%/2 mm.

**Figure 6:**
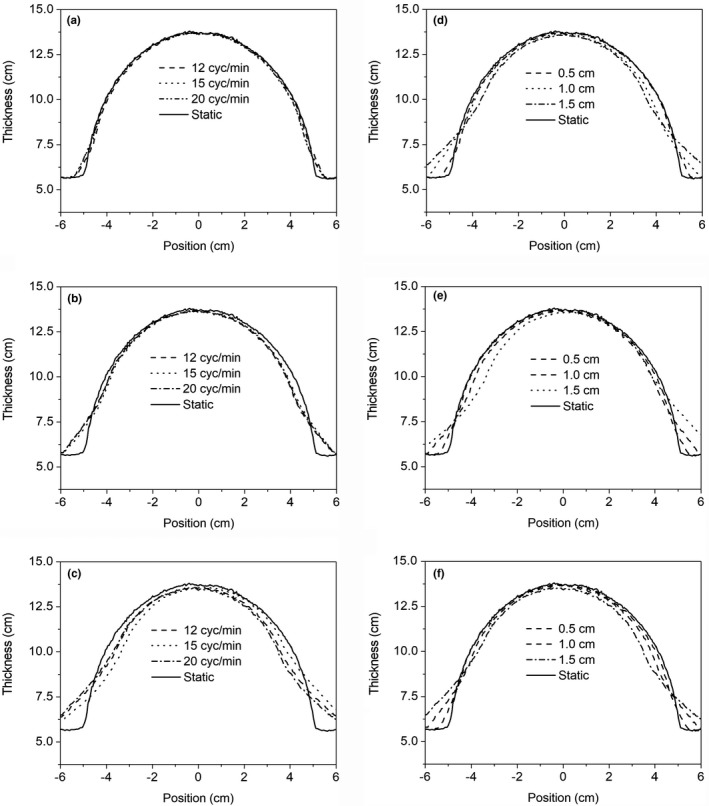
Comparison of one‐dimensional thickness profiles for fixed amplitude and different frequencies (a) amplitude = 0.5 cm (b) amplitude = 1.0 cm (c) amplitude = 1.5 cm and for fixed frequencies and different amplitudes (d) frequency = 12 cycles/min (e) frequency = 15 cycles/min, and (f) frequency = 20 cycles/min.

Figure 3 shows the 1D profiles through the thickness map for the static breast phantom, repeated three times. The percentage difference between these profiles is less than 1%, which is consistent with previous work and gives us confidence in the reproducibility of the quadratic calibration method.^3^ Figures 4(a)–4(c) shows the calibrated thickness maps of the phantom while undergoing linear sinusoidal motion for a frequency of 12 cycles/min and amplitudes of 0.5, 1.0, and 1.5 cm respectively. The thickness maps were converted to intensity, as shown in Section 2.D, and a 2D gamma analysis of the static vs moving phantom situations were performed. The results of the 2D gamma analysis comparing static vs moving phantom dose images for pixels within the ROI are shown in Figs. 4(d)–4(f) for a pass rate of 2%/2 mm. The effect of the motion, compared to the static case is shown, particularly in the periphery of the breast phantom, and the effect of the motion is more significant in the dose images as the amplitude of the motion is increased. Figures 5(a)–5(c) show the calibrated thickness maps for a motion amplitude of 1 cm and frequencies of 12, 15, and 20 cycles/min. The effect of a change in frequency for a particular amplitude of motion is less significant than the amplitude dependence shown in Fig. 4. Results of the 2D gamma analysis of the dose images for the motion sequences with different frequencies are shown in Figs. 5(d)–5(f) for pass criteria of 2%/2 mm. Figure 6 shows the 1D profiles across the thickness maps for the different magnitudes of sinusoidal motion. Figures 6(a)–6(c) are for motion amplitudes of 0.5, 1.0, and 1.5 cm respectively and three different frequencies for each amplitude. Figures 6(d)–6(f) are for motion frequencies of 12, 15, and 20 cycles/min respectively and three different amplitudes for each frequency.

**Figure 7:**
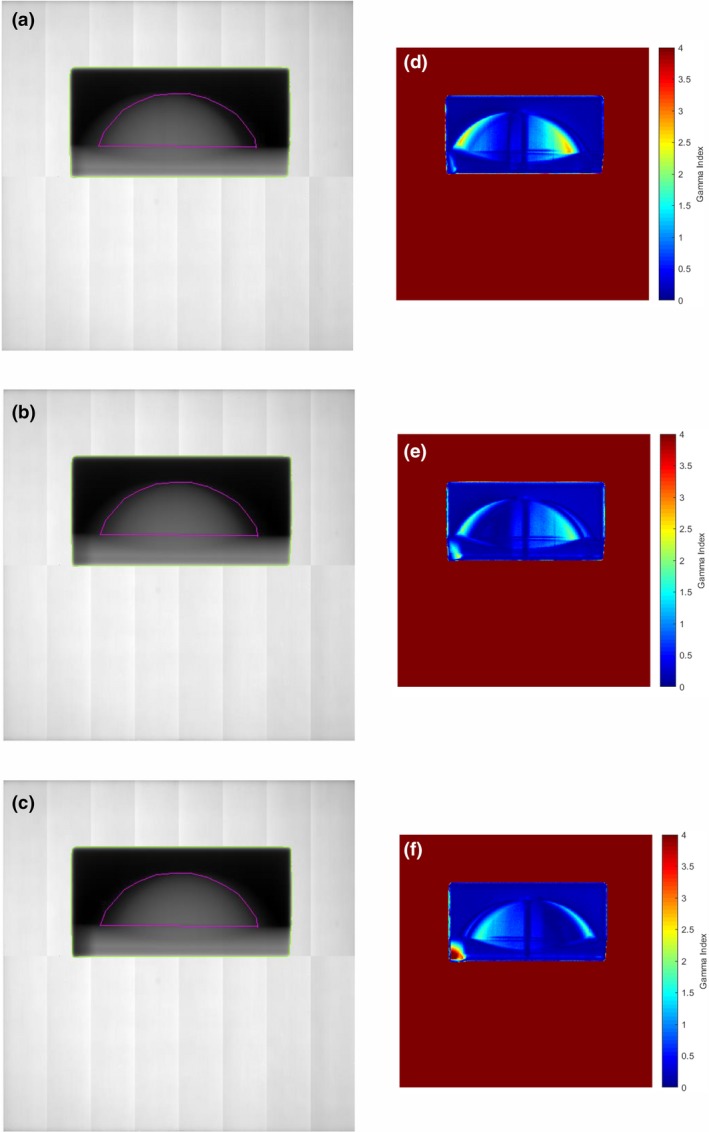
(a)–(c) Thickness maps for three (Patients 1, 4, and 5 in Table 2) of the patient motion sequences. Green line indicates the radiation field edge and magenta indicates the segmented “breast” region of interest. Results of a two‐dimensional gamma analysis of the target motion dose images determined from (a)–(c) compared to the reference static dose image are shown in (d)–(f) for gamma criteria of 3%/3 mm.

**Figure 8:**
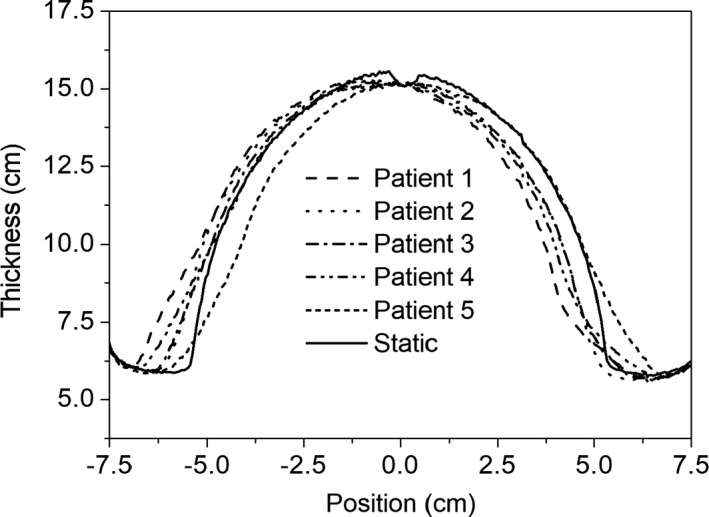
One‐dimensional profiles through the thickness maps for five different patient motion sequences.

Irradiation measurements were also performed on the breast phantom subject to the measured respiratory motion sequences of six patients. The CIRS motion platform with the breast phantom was programmed to reproduce the motion sequences of the patients and EPID measurements made during delivery of the treatment field. The calibrated 2D thickness maps for three of these patient motion sequences (patients 1, 4, and 5) are shown in Figs. 7(a)–7(c). The overlay of the contour of the breast from the static phantom shows how the motion influences the measured thickness image. The images show the average thickness of the breast tissue in each pixel over the duration of the delivery of the radiation field. The thickness images were converted back to intensity *I*(*x*,* y*), as described in Section 2.D, in order for the dose differences to be analyzed. The subsequent dose images of the phantom undergoing the different motions were compared with the static phantom dose images through a gamma analysis. The corresponding gamma analyses with pass criteria of 3%/3 mm, compared to the static breast phantom are shown in Figs. 7(d)–7(f). The 1D thickness profiles for five of the patient motion sequences (patients 1–5) are shown in Fig. 8 along with the static profile (solid line). The plots show the irregular asymmetric nature of the motion sequences.

Table 2 lists the results of an analysis of the dose differences within the “breast” ROI. The percentage of points within the ROI that have differences less than, 5%, 3%, 2%, and 1% are included in the table for the regular sinusoidal and irregular patient motion sequences. Table 3 lists the results of performing the gamma analysis of the static images compared to the moving phantom image of individual pixels within the segmented “breast” ROI. The table lists the percentage of pixels passing the Gamma Analysis (*γ* < 1.0) within the ROI for four different pass criteria, ranging from 5%/5 mm down to 1%/1 mm.

**Table 2:** Analysis of the dose differences, derived from the thickness images, between the static and moving phantom under different motion sequences. The percentage of pixels within the contoured “breast” region of interest with different levels of dose difference are listed

Motion		5%	3%	2%	1%
Amp (cm)	Freq (cpm)				
0.5	12	100	98.9	96.9	89.9
0.5	15	100	99.7	98.4	90.1
0.5	20	100	99.8	98.3	87.8
1	12	95.2	88.7	78.2	50.5
1	15	98	87.9	80.1	47
1	20	96.2	88	78.8	54.9
1.5	12	86.2	70.2	55.6	14.5
1.5	15	84.1	70.2	56.6	34.4
1.5	20	86.2	68.2	51.5	12.5
	Patient 0	85.3	75.5	66.3	57.3
	Patient 1	75.8	61.1	54.9	43.4
	Patient 2	84.5	71.3	59.7	49.4
	Patient 3	89.4	75.6	57.9	47.3
	Patient 4	79.8	62.3	52.1	41.2
	Patient 5	77.6	60.1	51.1	35.9

**Table 3:** Gamma analysis comparison of the dose images derived from the thickness images for the static and moving phantom under different motion sequences. Gamma analysis was only performed over the pixels within the contoured “breast” region of interest

Motion		Gamma analysis pass rate (%)			
Amp (cm)	Freq (cpm)	5%/5 mm	3%/3 mm	2%/2 mm	1%/1 mm
0.5	12	100.0	100.0	100.0	98.8
0.5	15	100.0	100.0	100.0	98.5
0.5	20	100.0	100.0	100.0	97.7
1	12	100.0	99.2	95.0	74.1
1	15	100.0	100.0	96.5	77.3
1	20	100.0	99.7	96.0	77.2
1.5	12	100.0	94.0	81.7	41.0
1.5	15	98.6	89.1	77.7	50.3
1.5	20	99.6	91.1	76.7	34.0
1.5	18	100.0	94.2	80.1	21.6
	Patient 0	98.	86.0	72.3	37.9
	Patient 1	88.9	68.2	46.9	24.7
	Patient 2	100.0	90.0	66.7	34.8
	Patient 3	100.0	92.2	75.2	45.9
	Patient 4	98.1	83.2	64.0	45.9
	Patient 5	99.5	85.7	68.5	49.1

## 4 DISCUSSION

In this paper we have investigated the effect of respiratory type motion on EPIDs calibrated for thickness using the quadratic calibration technique. The method used in this work was originally implemented to improve the image quality of megavoltage EPID images. The method removes detector gain variations and nonlinearities, systematic field flatness, and spectral variations that all degrade image quality. Random fluctuations in linear accelerator output remain, however, these are a few things one would want to monitor as part of an *in vivo* patient treatment verification process. As well as these image enhancement properties of the quadratic calibration method it has previously been shown that the method can also be used as a quantitative method for verification of the delivered radiotherapy treatment.^3^

The experimental results shown in Figs. 4, 5, and 6 for regular sinusoidal motions of different amplitudes and frequency indicate a more significant sensitivity to different amplitudes of motion rather than frequency. This is expected, as the number of cycles of motion over the duration of the radiation delivery is large and synchronization or so‐called interplay effects are not relevant for these static fixed collimation radiation deliveries, even at the lowest frequency used in this work.

The thickness maps show the average thickness of the breast phantom over the course of the irradiation. The effect of regular sinusoidal motion with increasing amplitudes on the average thickness of the breast is shown in Fig. 4. Figure 4(c) shows the thickness map when the phantom is moving with an amplitude of 1.5 cm. The overlay of the contour of the breast, obtained from the static phantom thickness map, shows the qualitative effect of the motion on the measured 2D thickness distribution of the breast “tissue”. Figure 6(c) shows the 1D profiles for the 1.5 cm motion, illustrating the quantitative effect of the motion on the measured dose at the EPID. The average thickness is lower for most of the profile except in the periphery.

Further quantitative analysis of the dose differences, shown in Table 2 indicates differences becoming more significant as the amplitude is increased. The most significant motion, with an amplitude of 1.5 cm, results in 85% of the pixels within the “breast” ROI having a dose difference of 5% or less. For motion with an amplitude of 1 cm approximately 80% of the pixels within the same ROI have a dose difference of 2% or less. The agreement improves significantly for the smallest amplitude motion of 0.5 cm, 90% of the pixels within the ROI have a dose difference of 1% or less. Gamma analysis is commonly used to compare dose distributions in 1D, 2D, and three‐dimensional (3D) and is most valuable in situations where there are large dose gradients. This is the situation for the breast phantom and the reason for performing a gamma analysis. The gamma analysis plots in Figs. 4 and 5 indicate the differences are most significant in the periphery of the ROI. These are regions where the thickness is small such that motion will introduce more significant relative changes in thickness or separation of the phantom. The result will be significant changes in the dose at the EPID plane.

The qualitative changes in the 2D thickness maps for the case of patient motion, compared to the contour of the static phantom, can be seen in Figs. 7(a)–7(c) and show the asymmetric nature of the patient motion. The corresponding 2D gamma maps in Figs. 7(d)–7(f) show the regions where dose differences are greater than 3%/3 mm. Table 3 shows that all six patient motion sequences would result in EPID data where less than 90% of the points within the ROI pass the 3%/3 mm gamma criterion, a benchmark commonly used when comparing dose distributions in a clinical setting. Patient 1 has the most significant differences which is evident from the thickness map in Fig. 7(a) and 2D gamma map in Fig. 7(d). There would appear to be a baseline shift in the phantom, which would probably be corrected through daily image guidance prior to treatment delivery. However, this does show the method to be able to detect baseline shifts and motion that could occur during the treatment delivery and after pretreatment image guided setup verification.

The use of other motion tracking techniques such as external optical markers or surface monitoring^31^, ^32^ could also be used to provide further frequency information on the intra‐fraction motion to complement the EPID‐based equivalent thickness information.

We propose that this method will be of use for *in vivo* quantitative verification of the delivery of the patient treatment for different delivery techniques, a variation of the EPID dosimetry technique. Firstly, it can be used for conformal treatments, as shown in this paper, where the patient is free breathing during delivery. The thickness map measured during treatment can be used to verify that the breathing cycle of the patient has not deviated from that at the planning stage. For clinical treatments there would of course be no “static” reference image, instead we propose the use of a “day one” image or alternatively a predicted EPID image. We have previously shown how a Monte‐Carlo EPID image can be calculated using CT data of the phantom or patient.^3^ For treatment sites where motion may be significant an average 4DCT image could be used for the Monte‐Carlo EPID image prediction. Secondly, there is increasing use of breath‐hold techniques for breast and stereotactic lung treatments to reduce the effects of respiratory motion during treatments. EPID‐based thickness measurements can be used for verification that the breath‐hold position of the patient is reproduced at each fraction. Thirdly, the method would have use for verification of gated treatment deliveries. The thickness map of the ideal gated treatment would be expected to closely match that of the static case. A measured thickness map obtained during the gated treatment would therefore enable the verification of the accuracy of the gating process. This requires further investigation. Partial breast radiotherapy techniques could also be verified using this method. It also has been shown the technique can be used for verification of the patient position during IMRT treatments^14^, ^15^ and we are currently investigating its further use for IMRT and VMAT treatments techniques. This previous work also demonstrated the sensitivity of the method to delivery errors caused by multi‐leaf collimator positioning and the dose delivered per IMRT segment. It is also worth noting that EPID‐based *in vivo* dosimetry techniques are effectively collecting information for free, measuring and obtaining information from the beam exiting the patient that would otherwise be lost, and using technology that is readily available on most linear accelerators.

## 5 CONCLUSIONS

We have shown the effects of respiratory motion on thickness images generated from measured EPID data. Understanding how changes in delivered dose and patient anatomy manifest in the thickness images is important for the clinical use of this equivalent thickness method and for *in vivo* EPID dosimetry in general. The results have shown how the method is sensitive to changes in motion amplitude and different forms of individual patient motion as measured using the breast phantom. The EPID‐based equivalent thickness method could be combined with other methods to provide fraction‐by‐fraction information on patient motion and compliance with motion management techniques. This will increase confidence in the delivered treatment allowing new technologies to be used to further push the boundaries of precision and accuracy in radiotherapy.

## CONFLICTS OF INTEREST

The authors declare no conflict of interest.
